# Comparison of Retrospective and Prospective Falls Reporting Among Community-Dwelling Older People: Findings From Two Cohort Studies

**DOI:** 10.3389/fpubh.2021.612663

**Published:** 2021-03-12

**Authors:** Muhammad Hibatullah Romli, Lynette Mackenzie, Pey June Tan, Re On Chiew, Shun Herng Tan, Maw Pin Tan

**Affiliations:** ^1^Department of Rehabilitation Medicine, Faculty of Medicine and Health Sciences, Universiti Putra Malaysia, Serdang, Malaysia; ^2^Malaysian Research Institute on Ageing (MyAgeing^TM^), Universiti Putra Malaysia, Serdang, Malaysia; ^3^Discipline of Occupational Therapy, Faculty of Health Sciences, University of Sydney, Camperdown, NSW, Australia; ^4^Health Services and Policy Research Division, Geriatric Education and Research Institute, Singapore, Singapore; ^5^Faculty of Medicine, University of Malaya, Kuala Lampur, Malaysia

**Keywords:** third world, falls, developing countries, culture, aged

## Abstract

**Background:** While prospective recording is considered as the gold standard, retrospective recall is widely utilized for falls outcomes due to its convenience. This brings about the concern on the validity of falls reporting in Southeast Asian countries, as the reliability of falls recall has not previously been studied. This study aimed to evaluate the reliability of retrospective falls recall compared to prospective falls recording.

**Methods:** A secondary analysis of data from two prospective recording methods, falls diary and falls calendar, from two different research projects were obtained and analyzed. Retrospective falls recall was collected either through phone interview or follow-up clinic by asking the participants if they had fallen in the past 12 months.

**Results:** Two-hundred-sixty-eight and 280 elderly participated in the diary and calendar groups, respectively. Moderate (46%) and poor (11%) return rates were found on completed diary and calendar recording. Under-(32%) and overreporting (24%) of falls were found in diary compared to only 4% of overreporting for the calendar. Retrospective recall method achieved 57% response rate for the diary group (followed up at clinic) and 89% for the calendar group (followed up via telephone interview). Agreement between retrospective and prospective reporting was moderate for the diary (kappa =0.44; *p* < 0.001) and strong for the calendar (kappa = 0.89; *p* < 0.001).

**Conclusion:** Retrospective recall is reliable and acceptable in an observation study within healthy community older adults, while the combination of retrospective and prospective falls recording is the best for an intervention study with frailer older population. Telephone interview is convenient, low cost, and yielded a high response rate.

## Introduction

Falls among older people may lead to negative consequences to psychological and physical health, functional status, and increased mortality (1). While numerous studies have recorded a large number of risk factors for falls in older people, the presence of the history of at least one fall in the preceding year is the strongest predictor for the occurrence of subsequent falls (2). Therefore, it is important for healthcare practitioners to be able to correctly identify whether a previous fall had occurred.

It has been advocated that the prospective recording of falls represents the most accurate method of capturing actual incidences of falls, in terms of time and location of falls and the circumstances leading to a fall occurring. Recommended methods for prospective recording falls include telephone calls, diaries, calendars, and post-cards (3), with a minimum frequency of monthly recording (4). However, the use of prospective falls recording may have several drawbacks; the process is time consuming, requires a high level of commitment from health practitioners and clients, costly, and is susceptible to logistic issues such as inadvertently misplacing the records. Furthermore, missing data may occur due to non-returns.

The major challenges involved in the collection of prospective falls outcomes have led to researchers using retrospective recall of fall occurrences and number of falls as their selected method of measuring falls. Retrospective recall is achieved by asking the person about any previous falls occurrences on the common time frames of 6 or 12 months (3–5). While retrospective falls recording is a convenient, time-efficient, and low-cost method of identifying falls, the accuracy of the information obtained has often been criticized. Recall bias is prevalent among older adults. Cognitive impairment is common among older adults, with dementia being a major risk factor for falls, which lead to difficulty remembering the fall. Retrospective falls recall is therefore considered a less accurate method compared to prospective recall (4, 6). Moreover, cultural issues may also affect falls recall among Asian communities, who may view falls as part of normal aging, a natural event and unavoidable (7, 8).

Published guidelines have suggested that retrospective self-report may have resulted in over- and underreporting of falls (9). A scoping review on published studies on falls conducted in the Southeast Asian region suggested that all studies published thus far have only utilized retrospective falls recall to record fall events (5). In addition, an update review on falls studies in this region found that no prospective studies have been conducted. Accurate recording of falls is vital to form evidence-based policies for fall prevention in the region.

The recommendations favoring prospective recording of falls are based on published studies conducted exclusively in developed nations and pre-dominantly Caucasian communities. There is currently, no published evidence on the feasibility and accuracy of the various methods of recording falls in developing countries, and non-Caucasian populations. However, there is a need to identify appropriate methods to capture fall incidents among older people in this geographical region. This paper aims to evaluate the reliability of prospective and retrospective methods of recording falls among community-dwelling older people in Malaysia.

## Method

This paper involves analyses of secondary data from two different cohort studies. The studies had implemented differing methods of prospective data collection. The detail procedures of the studies have been reported elsewhere—the Malaysian Falls Assessment and Intervention Trial (MyFAIT) (10) and Malaysian Elders Longitudinal Research (MELoR) (11) prospective studies. In this paper, emphasis is given to the method of reporting falls outcomes for both cohorts. Data were retrieved on the characteristics of participant in both cohorts are on basic demographic information such as age, gender, ethnicity, education level, and living status. While other information that is valuable on falls such as body mass index (BMI), timed up and go (TuG), fear of falling status, and quality of life measured by CASP-19 instrument were also retrieved.

### Malaysian Falls Assessment and Intervention Trial

#### Design and Sampling

The MyFAIT involved community-dwelling older people at high risk of falls participating in an experimental two-armed randomized controlled trial (10). The MyFAIT study recruited a total of 268 older participants, aged 65 years and over, with two or more falls or one injurious fall in the preceding 12 months, into both arms of the study. Individuals with dementia, severe physical disabilities and psychiatric illnesses, or brain damage were excluded. All participants were recruited from primary care, the geriatric clinic, and the accident and emergency department.

#### Procedure

Participants were given monthly fall diaries with daily entries upon completion of their baseline assessment. New diaries were mailed to their homes before the end of every month with pre-paid envelopes to facilitate return of the previous month's completed diaries. Telephone reminders to return the diaries were made when no diaries were received after three consecutive months.

**Figure 1:**
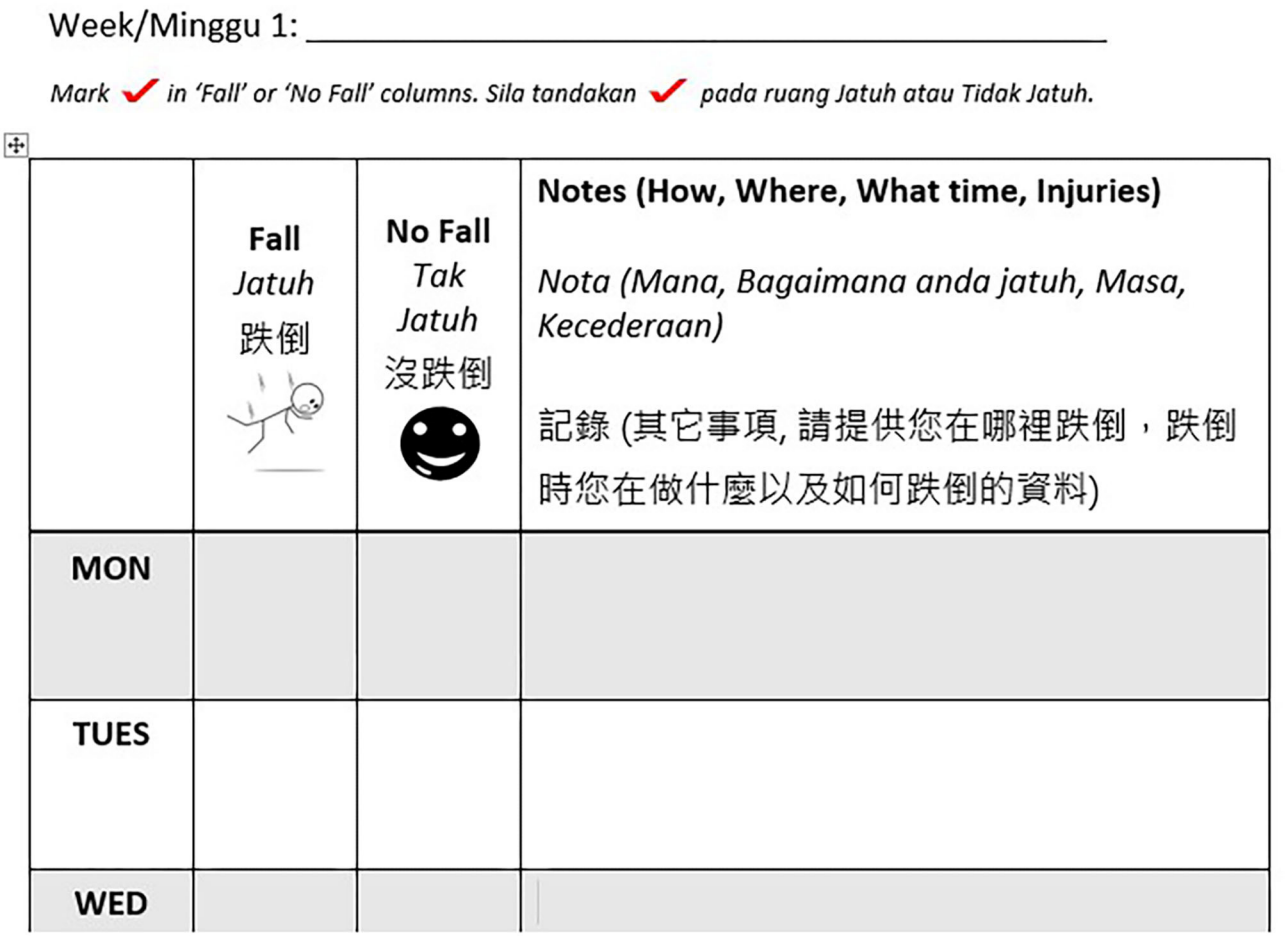
A sample of the monthly diary with daily entries used in Malaysian Falls Assessment and Intervention Trial (MyFAIT). The top row contains the date completed by the researcher before posting the diaries out to the participants. The participant is required to complete the diary at the end of the day, by marking the column “fall” or “no fall,” next to the correct day of the week. The wider column is completed with free text if the older person is able to write or with the assistance of a friend or relative and is used to describe the mechanism, time, location, and injury associated with a “fall”.

The MyFAIT fall diary (Figure 1) was designed and tailored to suit the heterogenous older Malaysian population in terms of language and educational attainment. The diary included written instructions in the three main languages used in Malaysia with daily entries prompting the individual to record the presence of any fall occurrence daily and to include descriptions of their fall. Free text space was used to record description of falls instead of tick boxes to reduce decision points and incorrect completion of data (12). The addition of pictures in the diaries ensured the inclusion of vulnerable groups of older adults who were illiterate or had lower levels of education. Visually impaired older adults received customized diaries with larger font sizes, and participants who preferred electronic diaries were emailed the softcopy versions. Two geriatricians evaluated the diaries for ease of use and accuracy of falls reporting. Support for diary completion were printed instructions on how to use the diary (12), along with contact details of the research assistant. These instructions were also repeated by the geriatrician during baseline assessment visits.

At the end of the study period of 12 months, participants were invited to visit the hospital for clinic follow-up. They were asked during their follow-up visit whether they had experienced at least one fall during their follow-up.

### Malaysian Elders Longitudinal Research Study

#### Design and Sampling

In brief, the MELoR project is a cohort study involving community-dwelling older people selected through simple random sampling stratified by the three major ethnicities of Malay, Chinese, and Indian from electoral rolls of three parliamentary constituencies in Greater Kuala Lumpur (12). In brief, the MELoR study had minimal exclusion criteria to maximize representativeness of valuable data on multidimensional aspects of health, economics, home design, ergonomics, media usage, technological engagement, physical activity, and legal issues among older adults. Only older people who refused to participate or unable to communicate or answer questions due to advanced dementia or severe speech impediments were excluded. For the purpose of this study, a total of 280 older participants of the MELoR project were randomly selected with simple random sampling from the overall cohort (i.e., *n* = 1489).

#### Procedure

**Figure 2:**
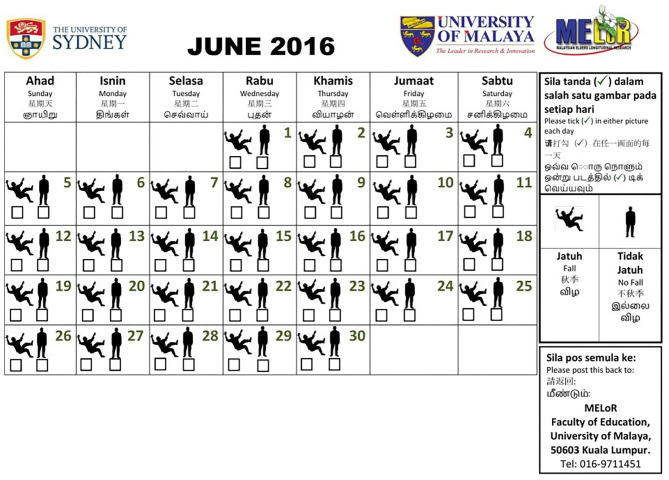
A sample of the falls calendar used in the Malaysian Elders Longitudinal Research (MELoR) study. A 1-month-to-a-page desktop calendar was posted to selected participants. The calendar prompted daily tick-box entries below a falling or standing silhouette indicating the presence and absence of a fall. The instructions were available in four common languages within the page, alongside contact details and the return address.

An attractive 1-month-to-a-page, 1-year calendar with daily entry was posted to all selected individuals as a desktop calendar for personal use, decorated with institutional and study logos and an attractive photograph of the study location as its front page. This was accompanied by a letter written in the four common languages used by older persons in Malaysia (English, Bahasa Melayu, Traditional Mandarin Chinese, and Tamil) to explain the purpose of the study and to provide a contact point for further clarification, as well as a self-addressed envelope to return the diary at the end of the study. The instructions to complete the calendar, in all four languages, were included within each page of the calendar. Each calendar page contained the dates of the month with one box dedicated to each day. Each box contained silhouettes of persons falling and standing with a tick box under each silhouette (Figure 2). The older person is required to tick on the relevant box each day, indicating whether they had fallen or not that day.

All participants were contacted at the end of the study to remind them to return their falls calendars and to ask them whether they had fallen in the previous year. A further history on the total number of falls were then obtained if the participant reported that they had fallen.

### Analysis

All statistical analyses were conducted using the Statistical Package for Social Sciences (SPSS) version 22. Basic demographic data were analyzed using descriptive statistics of frequencies and percentages for categorical data and means with standard deviations for continuous data. Comparison on the participants' characteristics between returners and non-returners of prospective recoding were also performed using inferential statistics such as independent *t*-test and chi-square depending on the nature of the variable. Response rates on retrospective recall and completion rates on prospective recordings were also analyzed using descriptive analysis. Interclass agreement between retrospective and prospective outcome was analyzed using Cohen's kappa agreement (13).

## Result

### Participant Characteristics and Response Rate

The characteristics of participants of the MyFAIT and the MELoR studies are summarized as follows. The MyFAIT participants were older (mean = 75.3; SD = 7.2) compared to the MELoR participants (mean = 71.9; SD = 8.98). The MyFAIT has marginally more women (67%) compared to the MELoR study (55%). As the MELoR study involved stratified sampling according to ethnicity, the ethnic composition of the MELoR study involved equal distributions for the three main ethnicities where Chinese contributed for 37.8% followed by Indian (32.1%), Malay (29.0%), and others (1.1%). While MyFAIT participants were mostly ethnic Chinese (61.9%), followed by Indian (19.0%), Malay (16.8%), and others (2.2%). The characteristics between the returners and non-returners on the two cohorts are relatively similar (*p*_*s*_ > 0.05) except in the MyFAIT cohort where more Chinese ethnicity (*p* = 0.003) and better CASP-19 score (*p* = 0.015) were identified among returners compared to non-returners. Table 1 describes in detail the characteristics of the returners and non-returners of the prospective recordings in the two cohorts.

**Table 1:** Characteristics of the returners and non-returners of prospective recordings.

**Characteristics**	**MyFAIT (*****N*** **=** **268)**		***p*-value**	**MELoR (*****N*** **=** **280)**		***p*-value**
	**Returners (*n* = 193; 72.1%)**	**Non-returners (*n* = 75; 27.9%)**		**Returners (*n* = 31; 11.1%)**	**Non-returners (*n* = 249; 88.9%)**	
**Prospective recording**	193 (100%)	75 (100%)		31 (100%)	249 (100%)	
Having falls	82 (42.5%)	–		5 (16.1%)	–	
No fall	111 (57.5%)	–		26 (83.9%)	–	
**Retrospective recall**	127 (65.8%)	25 (33.3%)		31 (100%)	204 (81.9%)	
Having falls	55 (43.3%)^a^	9 (36.0%)^a^	0.499^◇^	6 (19.4%)^a^	30 (14.7%)^a^	0.556^◇^
No fall	72 (56.7%)^a^	16 (64.0%)^a^		25 (80.6%)^a^	174 (85.3%)^a^	
**Age [mean (SD)]**	74.9 (7.03)	76.3 (7.72)	0.166^†^	67.5 (8.83)	69.1 (8.98)	0.381^†^
**Gender**						
Male	63 (32.6%)	24 (32.0%)	0.920^◇^	15 (48.4%)	109 (43.8%)	0.626^◇^
Female	130 (67.4%)	51 (68.0%)		16 (51.6%)	140 (56.2%)	
**Ethnicity**						
Malay	30 (15.3%)	15 (20.8%)	0.008^*^^◇^	7 (22.6%)	74 (29.7%)	0.363^◇^
Chinese	130 (67.4%)	36 (48.0%)		16 (51.6%)	89 (35.7%)	
Indian	28 (14.3%)	23 (31.9%)		8 (25.8%)	83 (33.3%)	
Others	5 (2.6%)	1 (1.4%)		0	3 (1.3%)	
**Education level**						
None	NA	NA	NA	0	6 (2.5%)	0.602^◇^
Primary	NA	NA		4 (12.9%)	52 (20.9%)	
Secondary	NA	NA		13 (41.9%)	95 (38.3%)	
Vocational/tertiary	NA	NA		14 (45.2)	95 (38.3%)	
**Living alone**						
Yes	138 (71.5%)	54 (72.0%)	0.910^◇^	2 (6.5%)	22 (8.8%)	0.655^◇^
No	55 (28.5%)	21 (28.0%)		29 (93.5%)	227 (91.2%)	
**Fear of falling**						
Yes	123 (63.7%)^b^	54 (72.0%)^b^	0.130^◇^	17 (58.6%)^c^	183 (74.4%)^c^	0.071^◇^
No	70 (36.3%)^b^	21 (28.0%)^b^		12 (41.4%)^c^	63 (25.6%)^c^	
**BMI [mean (SD)]**	23.9 (3.98)	25.3 (4.38)	0.014^†^	24.3 (4.06)	25.6 (4.49)	0.139^†^
**TUG [mean (SD)]**	17.2 (12.99)	19.3 (9.69)	0.228^†^	11.5 (3.54)	12.6 (3.94)	0.122^†^
**CASP-19 total [mean (SD)]**	43.5 (9.59)	40.1 (11.36)	0.015^*^^†^	43.5 (7.47)	43.9 (7.55)	0.805^†^
CASP-19 Control [mean (SD)]	8.1 (2.86)	7.5 (3.31)	0.209^†^	7.7 (2.85)	7.8 (2.73)	0.868^†^
CASP-19 Autonomy [mean (SD)]	11.9 (2.82)	10.7 (3.09)	0.003^*^^†^	10.9 (2.61)	10.9 (2.89)	0.985^†^
CASP-19 Pleasure [mean (SD)]	13.3 (2.73)	12.6 (3.69)	0.139^†^	13.7 (1.73)	13.7 (2.02)	0.905^†^
CASP-19 Self-realization [mean (SD)]	10.3 (3.64)	9.3 (4.16)	0.062^†^	11.2 (2.66)	11.5 (2.55)	0.507^†^

◇ *Independent t-test*.

† *Chi-square*.

* *p ≤ 0.05*.

a *Compared with the total valid retrospective sample*.

b *Based on the FES-I questionnaire*.

c *Based on a single-item question of “Are you afraid of falling?”*.

*NA, data are not available*.

In the 12-month study period of MyFAIT, 193 (72%) of the 268 participants had returned at least one diary. In further detail, 1,488 individual diaries (46%) were collected out of a possible 3,216. One hundred fifty-two (57%) attended follow-up at 12 months. Of the 280 participants included in the MELoR study, 31 (11%) completed the calendar. Fifteen (47%) of the 31 participants who returned their annual calendar did so spontaneously, and another 16 (53%) did so after receiving the reminder phone calls. Response rate for phone calls was 83.9% (*n* = 235).

### Fall Incidents

#### MyFAIT

At least one prospective fall was recorded in 82 (42.5%) of the 193 participants who have returned at least one monthly diary. One hundred fifty-two (56.7%) of the 268 participants attended follow-up assessments, with retrospective, self-reported falls in 64 (42.1%) of the 152 participants.

#### MELoR

Of the 31 falls calendar returned, 5 (16.1%) participants recorded at least one fall in the past 12 months. Of the 235 participants contacted by phone, fall incidents were recalled by 36 (15.3%) of the older participants for the past 12 months.

### Agreement Between Prospective and Retrospective Methods

Table 2 summarizes the level of agreement between these two methods.

**Table 2:** Agreement between prospective and retrospective falls recording.

	**MyFAIT (*n* = 127)**	**MELoR (*n* = 31)**
**Fall occurrences**		
Prospective reported	56 (44%)	5 (16%)
Retrospective	55 (43%)	6 (19%)
Agreement	91 (72%)	30 (97%)
Underreporting	18/56 (32%)	0/5 (0%)
Overreporting	17/71 (24%)	1/26 (4%)
Kappa agreement	0.44	0.89
*p*-value	<0.001	<0.001

#### MyFAIT

Agreement between the presence and absence of falls in both prospective records and retrospectively recall was 72% (*n* = 91/127). Fifty-six (44%) reported falls in their diaries, while 55 (43%) reported falls during their follow-up visits. Eighteen (32%) of the 56 fallers who had recorded falls in their diaries failed to report falls during their follow-up visit (underreporting). Conversely, 17 (24%) of the 71 who had not recorded falls in their diaries reported falls during their follow-up visit (overreporting). An interclass agreement using kappa analysis on retrospective self-reported falls and prospective falls diaries was moderate (*k* = 0.44; *p* = 0.001). Comparing the mean (standard deviation) number of falls, MyFAIT's prospective fall diaries recorded 0.97 (1.89) falls, and retrospective recall at 12 months reported 0.74 (1.14) falls.

#### MELoR

Of the 31 participants for whom retrospective and prospective records were available, agreement between the presence and absence of fall occurrence in prospective calendar records and retrospective recall was 97% (*n* = 30). Six participants (19%) reported falls during telephone follow-up, five (16%) of whom had actually recorded fall occurrences in their calendar. There was therefore no underreporting, as all fallers identified using falls calendar reported falls during their telephone follow-up. One of the 26 non-fallers who returned their calendars reported falls during their telephone follow-up, yielding an overreporting rate of 4% for telephone follow-up. An interclass agreement using kappa analysis yielded strong agreement between both calendar recording and telephone recall (*k* = 0.89; *p* < 0.001).

## Discussion

Accurate falls reporting is important to ensure reliable findings in falls research and help clinicians to identify older people at risk for recurrent falling and plan for future intervention and prevention efficiently. This current study indicates that there is an acceptable and satisfactory agreement between retrospective self-reported falls with prospective falls recording methods in both a hospital-based intervention study involving high risk falls and a community-based cohort study involving the general older population. Both prospective recording and retrospective recall yielded similar fall rates with a discrepancy of only one participant in both studies. If prospective falls recording was considered the gold standard, both over- and underestimations occurred with retrospective recall in MyFAIT, while minimal differences occurred in MELoR. This phenomenon is also reported in previous studies (4, 14–16). Nevertheless, the level of agreement between prospective and retrospective recording were within acceptable limits, with better agreement observed in a healthier, community-based cohort compared to individuals with recurrent or injurious falls in the intervention study.

Prospective recording of falls is markedly more demanding on human and financial resources and is prone to attrition (12). In resource poor settings, this may therefore not be a reasonable solution. Monthly fall diaries that requires monthly postal correspondence and phone calls to participants was not replicable in a large community-based cohort study. The alternative of a fall calendar yielded a low return rate, despite phone call reminders. The lack of engagement with prospective fall recording in the community cohort compared to the falls cohort may also have occurred as the falls cohort were incentivized by their involvement in a fall prevention intervention that may benefit them directly (17). Health-seeking behavior is likely to be higher for people with illness or injuries (18). The community cohort, on the other hand, did not receive incentives for returning their diaries and lacked the motivation of identifying falls since they had not experienced or identified falls as an issue. While incentives are important to reduce attrition in longitudinal studies, it was not always possible to ensure adequate reward with competing priorities of a cohort study. Healthier, older adults may also perceive falls as a natural occurrence and a trivial issue related to aging (7), reducing the likelihood of calendar returns. The frequency of the researchers contacting the participants for reminder purpose might have an impact on the adherence of the participants to return the diary. The MyFAIT study has higher frequency of contact compared to the MELoR study. Thus, the researchers had the opportunity to increase their effort to retrieve the diary as many as possible to explain on why the response rate is higher for the MyFAIT study. With increasing use of electronic means of communication, the role of postal services has decreased. Therefore, older persons may not have developed a habit of posting letters, with post-boxes not being accessible to them. Alternative strategies such as the use of Smartphone Apps are not practical solutions, as the smartphone use among the Malaysian older population remains limited (19) and the App requires resources for development and maintenance.

Response rates for retrospective evaluation were higher in the community cohort than in the falls intervention study. Hospital visits were meaningful to the intervention participants and appreciated, as they received medical attention required; however, some of the frailer participants in the falls cohort had died, were no longer contactable, or were too frail to visit the hospital and may have an impact on the low response rate. In the community cohort, limited resources and no requirement removed the possibility of hospital visits and, if conducted, would have unnecessarily inconvenienced participants. Hence, participants could only be followed up via telephone, but the response rate is much higher. The methods were, however, not comparable between studies due to the differences in participant characteristics. In addition, falls intervention study cohort consists more Chinese ethnicity who are older and have higher falls rate (11, 20). The ethnicity representation of more Chinese and Indian of this study compared to the national population is somewhat different (20). However, the reason is that the two cohort studies were conducted in an urban setting where a larger proportion of the older population were ethnic Chinese and Indian (20).

Despite issues with low calendar returns in the MELoR cohort and high follow-up attrition in the MyFAIT study, falls rates returned for both retrospective and prospective data collection were similar for both studies regardless of the method of detecting falls. The overall agreement between fall diaries and retrospective recall was moderate for MyFAIT, while calendar-recorded events and retrospective telephone recall for MELoR showed nearly perfect agreement. This implies that retrospective telephone recall should be the preferred method of detecting falls in our community cohort. However, this finding should be interpreted with caution due to the low calendar return rate for our community cohort leading to non-return bias, with individuals who remember to return diaries significantly more likely to remember their falls accurately. While the similar falls rates for both calendar records and telephone recall are reassuring, it does not remove the possibility that calendar returners were less likely to fall while the overall cohort underreported falls (21). Conversely, while falls rates are nearly identical between prospective diaries and retrospective recall in MyFAIT, the high attrition rates for retrospective recall should detract from the temptation to only use retrospective recall as the sole method of detecting falls. Diary exercises alone would yield a response rate ranging from 46 to 72% depending on definition. We found that combining both prospective reporting and retrospective recall during clinic visits yielded an acceptable overall response rate of 82%. Hence, a combination of monthly diary with self-addressed envelopes and end-of-study visit is our recommended approach to falls detection in intervention studies for secondary falls prevention.

Our findings somewhat contradicted to that with previous studies, where prospective recording is a better option (14–16), but similar to that of a recent study (21). Developing countries face challenges in terms of financial constraints, lack of infrastructure, and lack of understanding about research among study participants (22). The differences observed in our two studies could just as well be attributed to cultural diversities in perception of falls, language use, and level of education (7, 11, 23). Despite emphasis on falls detection, as a strong indicator for recurrent falls, clinicians should also be aware of the prevailing opinion that primary prevention, that is, identification of increased risk of falling before any incident fall, would be preferable. This latter approach could be aided by the use of standardized instruments and new technology (23–25). Our findings has established suitable methods for recording falls, which will help alleviate mistrust on falls data recording retrospectively in community cohorts and encourage much needed falls-related research in developing countries.

The difference in the number of Chinese is higher among returners than non-returners in the MyFAIT group. There is complex explanation for this situation, as a previous study indicates that Malay ethnicity has the initiative for first action for consulting physician about their health concern (18), and falls are highest among Indian but lowest among Malay (26). However, the Chinese are the most in initiating self-treatment (18). Perhaps, this may explain why the Chinese are mostly returning their calendar, as they will seek for any possible approach to resolve their sustained health issue. This study also finds that the older people are with higher quality of life score, especially on autonomy aspect. People with better quality of life are more prone to have better health behavior (27), and who has higher autonomy is more responsible about oneself and has control over one's own health (18). This is translated into higher return rate of the diary among older people with better quality of life and autonomy.

One critical limitation of this study is the low return rate for prospective recording especially on the MELoR study, which may affect the reliability of falls outcomes collected in this way. The high reliability in the MELoR study may also be due to volunteer bias; however, the impact of this is presumed not significant (28). However, with the low response rate, and the study population comprising relatively healthy community-dwelling older people in an urban area, the generalizability of the study to rural older folks and disease-specific populations, such as those with cognitive impairment, may be limited. Future studies should therefore target the latter populations to further establish the most suitable methods of detecting falls in these special populations.

Current clinical practice recommends the confirmation of the accuracy of falls history among older persons through collateral histories from the family or carers (29) or to extrapolate falls risk based on balance and mobility functions, sensory (i.e., vision), and medication intake (30). This study, however, suggests that practitioners should have confidence in self-reported or retrospective recall of falls among older patients, which should then go on to inform timely administration and early intervention of secondary prevention measures. The previous assumption that older people may conceal their falls (29, 31) may not necessarily be of concern based on our study findings. The Asian culture has strong respect and trust to professionals and expert with little inquiry on practice (32). Client–practitioner relationship is built on trust, and the information exchange is believed to enhance the quality of interventions (29, 31, 33).

## Conclusion

Retrospective falls recall using telephone interviewing in cohort studies involving community-dwelling older adults is preferred for measuring falls in our developing country setting, with low response rates likely for prospective recording methods. In addition, retrospective recall is the preferred method in developing countries due to its convenience and low cost. However, in intervention studies involving older fallers, a combination of prospective recording and retrospective recall should be considered. Our findings have established suitable methods for recording falls, which will help alleviate mistrust on falls data recording retrospectively in community cohorts and encourage much needed falls-related research internationally, especially in developing countries.

## Data Availability Statement

The data analyzed in this study is subject to the following licenses/restrictions: The raw data supporting the conclusions of this article will be made available by the authors, without undue reservation. Requests to access these datasets should be directed to mptan@ummc.edu.my.

## Ethics Statement

The studies involving human participants were reviewed and approved by Both, the MyFAIT trial (MEC Ref No: 943.21) and the MELoR project (MEC Ref No: 943.6) received ethical clearance from the University of Malaya. The patients/participants provided their written informed consent to participate in this study.

## Author Contributions

MR, LM, PT, RC, ST, and MT are equally contributed on designing and conducting the study and writing the manuscript. MR, LM, PT, and MT provides the critical feedback on the manuscript. All authors approved the final version of the manuscript.

## Conflict of Interest

The authors declare that the research was conducted in the absence of any commercial or financial relationships that could be construed as a potential conflict of interest.
